# Supplementary material for: Prevalence and richness of malaria and malaria-like parasites in wild birds from different biomes in South America
Source: PeerJ. 2022 May 19;10:e13485. doi: 10.7717/peerj.13485 (PMC9124460; doi:10.7717/peerj.13485)
Supplement: Supplemental Information 2 [file peerj-10-13485-s002.docx]

Supplementary Table 1- Number of wild birds examined and infected by *Plasmodium* spp./ *Haemoproteus* spp. from Cerrado.

| **Wild Birds Family/Species** | **Wild Birds Numbers Captured** | **Wild Birds Numbers Infected** |
| --- | --- | --- |
| **Alcedinidae** |  |  |
| *Chloroceryle aenea* (Pallas, 1764) | 3 | 1 |
| **Bucconidae** |  |  |
| *Monasa nigrifrons* (Spix, 1824) | 4 | 3 |
| *Nystalus chacuru* (Vieillot, 1816) | 1 | 1 |
| *Nystalus maculatus* (Gmelin, 1788) | 1 | 1 |
| **Caprimulgidae** |  |  |
| *Nyctidromus albicollis* (Gmelin, 1789) | 3 | 2 |
| **Cardinalidae** |  |  |
| *Cyanoloxia rothschildii* (Bartlett, 1890) | 3 | 3 |
| *Piranga flava* (Vieillot, 1822) | 1 | 0 |
| **Columbidae** |  |  |
| *Leptotila rufaxilla* (Richard & Bernard, 1792) | 1 | 1 |
| *Columbina talpacoti* (Temminck, 1810) | 2 | 1 |
| **Cuculidae** |  |  |
| *Crotophaga ani* (Linnaeus, 1758) | 2 | 2 |
| **Dendrocolaptidae** |  |  |
| *Dendrocincla fuliginosa* (Vieillot, 1818) | 1 | 0 |
| *Dendrocolaptes certhia* (Boddaert, 1783) | 1 | 1 |
| *Dendrocolaptes platyrostris* (Spix, 1825) | 1 | 0 |
| *Dendroplex picus* (Gmelin, 1788) | 5 | 2 |
| *Lepidocolaptes angustirostris* (Vieillot, 1818) | 3 | 0 |
| *Sittasomus griseicapillus* (Vieillot, 1818) | 5 | 4 |
| *Xiphorhynchus obsoletus* (Lichtenstein, 1820) | 1 | 1 |
| **Fringillidae** |  |  |
| *Euphonia chlorotica* (Linnaeus, 1766) | 1 | 0 |
| *Euphonia violacea* (Linnaeus, 1758) | 4 | 1 |
| **Furnariidae** |  |  |
| *Synallaxis albescens (*Temminck, 1823) | 1 | 1 |
| *Synallaxis gujanensis* (Gmelin, 1789) | 1 | 0 |
| **Galbulidae** |  |  |
| *Galbula ruficauda* (Cuvier, 1816) | 10 | 6 |
| **Hirundinidae** |  |  |
| *Progne chalybea* (Gmelin, 1789) | 3 | 1 |
| **Icteridae** |  |  |
| *Cacicus cela* (Linnaeus, 1758) | 9 | 8 |
| *Gnorimopsar chopi* (Vieillot, 1819) | 3 | 3 |
| **Melanopareiidae** |  |  |
| *Melanopareia torquata* (Wied, 1831) | 1 | 0 |
| **Mimidae** |  |  |
| *Mimus saturninus* (Lichtenstein, 1823) | 2 | 0 |
| **Parulidae** |  |  |
| *Basileuterus culicivorus* (Deppe, 1830) | 13 | 4 |
| *Myiothlypis flaveola* (Baird, 1865) | 3 | 1 |
| **Passerelidae** |  |  |
| *Ammodramus humeralis* (Bosc, 1792) | 1 | 0 |
| *Arremon taciturnus* (Hermann, 1783) | 4 | 3 |
| **Passeridae** |  |  |
| *Passer domesticus* (Linnaeus, 1758) | 1 | 1 |
| **Picidae** |  |  |
| *Colaptes melanochloros* (Gmelin, 1788) | 1 | 1 |
| *Melanerpes candidus* (Otto, 1796) | 1 | 0 |
| *Picumnus albosquamatus* (d'Orbigny, 1840) | 9 | 4 |
| **Pipridae** |  |  |
| *Antilophia galeata* (Lichtenstein, 1823) | 2 | 2 |
| *Machaeropterus pyrocephalus* (Sclater, 1852) | 1 | 0 |
| *Manacus manacus* (Linnaeus, 1766) | 21 | 14 |
| *Pipra fasciicauda (*Hellmayr, 1906) | 111 | 56 |
| **Platyrinchidae** |  |  |
| *Platyrinchus mystaceus* (Vieillot, 1818) | 3 | 1 |
| **Ramphastidae** |  |  |
| *Pteroglossus inscriptus* (Swainson, 1822) | 1 | 1 |
| **Rhynchocyclidae** |  |  |
| *Corythopis torquatus Tschudi, 1844* | 5 | 2 |
| *Hemitriccus margaritaceiventer (d'Orbigny & Lafresnaye, 1837)* | 9 | 3 |
| *Hemitriccus striaticollis* (Lafresnaye, 1853) | 8 | 2 |
| *Leptopogon amaurocephalus* (Tschudi, 1846) | 8 | 4 |
| *Mionectes oleagineus* (Lichtenstein, 1823) | 1 | 0 |
| *Poecilotriccus fumifrons* (Hartlaub, 1853) | 8 | 3 |
| *Tolmomyias flaviventris* (Wied, 1831) | 2 | 1 |
| *Tolmomyias poliocephalus* (Taczanowski, 1884) | 1 | 1 |
| **Thamnophilidae** |  |  |
| *Dysithamnus mentalis* (Temminck, 1823) | 7 | 3 |
| *Formicivora grisea* (Boddaert, 1783) | 10 | 6 |
| *Formicivora rufa* (Wied, 1831) | 11 | 3 |
| *Hypocnemoides maculicauda* (Pelzeln, 1868) | 6 | 1 |
| *Myrmotherula axillaris* (Vieillot, 1817) | 3 | 2 |
| *Myrmotherula multostriata* (Sclater, 1858) | 2 | 1 |
| *Sakesphorus luctuosus* (Lichtenstein, 1823) | 3 | 2 |
| *Taraba major* (Vieillot, 1816) | 2 | 2 |
| *Thamnophilus amazonicus* (Sclater, 1858) | 1 | 1 |
| *Thamnophilus doliatus* (Linnaeus, 1764) | 1 | 0 |
| *Thamnophilus torquatus* (Swainson, 1825) | 6 | 1 |
| **Thraupidae** |  |  |
| *Coereba flaveola* (Linnaeus, 1758) | 27 | 12 |
| *Coryphospingus pileatus* (Wied, 1821) | 13 | 9 |
| *Cyanerpes cyaneus* (Linnaeus, 1766) | 1 | 1 |
| *Dacnis cayana* (Linnaeus, 1766) | 6 | 2 |
| *Hemithraupis guira* (Linnaeus, 1766) | 3 | 3 |
| *Neothraupis fasciata* (Lichtenstein, 1823) | 1 | 1 |
| *Ramphocelus carbo* (Pallas, 1764) | 12 | 9 |
| *Saltatricula atricollis* (Vieillot, 1817) | 2 | 2 |
| *Saltator maximus* (Statius Muller, 1776) | 9 | 5 |
| *Schistochlamys ruficapillus* (Vieillot, 1817) | 8 | 7 |
| *Sporophila angolensis* (Linnaeus, 1766) | 1 | 1 |
| *Sporophila plumbea* (Wied, 1830) | 1 | 1 |
| *Lanio cristatus* (Linnaeus, 1766) | 4 | 3 |
| *Lanio luctuosus* (d'Orbigny & Lafresnaye, 1837) | 1 | 1 |
| *Tachyphonus rufus* (Boddaert, 1783) | 5 | 3 |
| *Tangara cayana* (Linnaeus, 1766) | 8 | 2 |
| *Thlypopsis sordida* (d'Orbigny & Lafresnaye, 1837) | 6 | 2 |
| *Tangara palmarum* (Wied, 1821) | 4 | 0 |
| *Tangara sayaca* (Linnaeus, 1766) | 2 | 0 |
| *Volatinia jacarina* (Linnaeus, 1766) | 25 | 9 |
| **Tityridae** |  |  |
| *Pachyramphus polychopterus* (Vieillot, 1818) | 2 | 0 |
| **Troglodytidae** |  |  |
| *Cantorchilus leucotis* (Lafresnaye, 1845) | 5 | 5 |
| *Pheugopedius genibarbis* (Swainson, 1838) | 10 | 1 |
| *Troglodytes musculus* (Naumann, 1823) | 2 | 0 |
| **Trogonidae** |  |  |
| *Trogon curucui* (Linnaeus, 1766) | 1 | 1 |
| **Turdidae** |  |  |
| *Turdus amaurochalinus* (Cabanis, 1850) | 1 | 0 |
| *Turdus leucomelas* (Vieillot, 1818) | 22 | 19 |
| **Tyrannidae** |  |  |
| *Camptostoma obsoletum* (Temminck, 1824) | 5 | 1 |
| *Casiornis fuscus* (Sclater & Salvin, 1873) | 1 | 0 |
| *Cnemotriccus fuscatus* (Wied, 1831) | 4 | 1 |
| *Elaenia chiriquensis* (Lawrence, 1865) | 28 | 14 |
| *Elaenia cristata* (Pelzeln, 1868) | 27 | 11 |
| *Elaenia flavogaster* (Thunberg, 1822) | 8 | 3 |
| *Elaenia mesoleuca* (Deppe, 1830) | 1 | 1 |
| *Elaenia parvirostris* (Pelzeln, 1868) | 2 | 0 |
| Elaenia spectabilis (Pelzeln, 1868) | 1 | 0 |
| *Empidonomus varius* (Vieillot, 1818) | 1 | 0 |
| *Euscarthmus meloryphus* (Wied, 1831) | 3 | 1 |
| *Euscarthmus rufomarginatus* (Pelzeln, 1868) | 1 | 1 |
| *Inezia subflava* (Sclater & Salvin, 1873) | 1 | 0 |
| *Lathrotriccus euleri* (Cabanis, 1868) | 6 | 1 |
| *Megarynchus pitangua* (Linnaeus, 1766) | 1 | 1 |
| *Myiarchus ferox* (Gmelin, 1789) | 3 | 1 |
| *Myiarchus swainsoni* (Cabanis & Heine, 1859) | 7 | 1 |
| *Myiarchus tuberculifer* (d'Orbigny & Lafresnaye, 1837) | 1 | 0 |
| *Myiopagis caniceps* (Swainson, 1835) | 1 | 0 |
| *Myiopagis gaimardii* (d'Orbigny, 1839) | 10 | 6 |
| *Myiopagis viridicata* (Vieillot, 1817) | 6 | 1 |
| *Myiophobus fasciatus* (Statius Muller, 1776) | 4 | 2 |
| *Myiozetetes cayanensis* (Linnaeus, 1766) | 3 | 2 |
| *Phaeomyias murina* (Spix, 1825) | 7 | 1 |
| *Ramphotrigon ruficauda* (Spix, 1825) | 1 | 0 |
| *Serpophaga subcristata* (Vieillot, 1817) | 2 | 1 |
| *Sublegatus modestus* (Wied, 1831) | 1 | 0 |
| *Tyrannus albogularis* (Burmeister, 1856) | 1 | 0 |
| *Tyrannus melancholicus* (Vieillot, 1819) | 2 | 1 |
| **Vireonidae** |  |  |
| *Cyclarhis gujanensis* (Gmelin, 1789) | 4 | 3 |
| *Hylophilus pectoralis* (Sclater, 1866) | 4 | 3 |
| *Vireo olivaceus* (Linnaeus, 1766) | 7 | 6 |
| **Xenopidae** |  |  |
| *Xenops minutus* (Sparrman, 1788) | 1 | 0 |
| **Total** | 676 | 331 |
