# Supplementary material for: Prevalence and richness of malaria and malaria-like parasites in wild birds from different biomes in South America
Source: PeerJ. 2022 May 19;10:e13485. doi: 10.7717/peerj.13485 (PMC9124460; doi:10.7717/peerj.13485)
Supplement: Supplemental Information 3 [file peerj-10-13485-s003.docx]

| **Wild Birds Family/Species** | **Wild Birds Numbers Captured** | **Wild Birds Numbers Infected** |
| --- | --- | --- |
| **Cardinalidae** |  |  |
| *Saltator orenocensis* (Lafresnaye, 1846) | 13 | 3 |
| **Emberizidae** |  |  |
| *Tiaris bicolor* (Linnaeus, 1766) | 141 | 30 |
| **Icteridae** |  |  |
| *Icterus nigrogularis* (Hahn, 1816) | 19 | 19 |
| *Icterus icterus* (Linnaeus, 1766) | 6 | 3 |
| **Fringillidae** |  |  |
| *Cardinalis phoeniceus* (Bonaparte, 1838) | 80 | 59 |
| **Furnariidae** |  |  |
| *Synallaxis candei* (d'Orbigny & Lafresnaye, 1837) | 6 | 0 |
| *Xiphorhynchus picus* (Gmelin, 1788) | 43 | 15 |
| **Mimidae** |  |  |
| *Mimus gilvus* (Vieillot, 1808) | 51 | 43 |
| **Picidae** |  |  |
| *Melanerpes rubricapillus* (Cabanis, 1862) | 15 | 11 |
| **Psittacidae** |  |  |
| *Amazona barbadensis* (Gmelin, 1788) | 20 | 0 |
| *Aratinga pertinax* (Linnaeus, 1758) | 11 | 8 |
| **Thamnophilidae** |  |  |
| *Formicivora intermedia* (Boddaert, 1783) | 4 | 1 |
| *Sakesphorus canadenses* (Linnaeus, 1766) | 7 | 5 |
| **Thraupidae** |  |  |
| *Coereba flaveola* (Linnaeus, 1758) | 18 | 0 |
| *Coryphospingus pileatus* (Wied, 1821) | 4 | 4 |
| *Saltator coerulescens* (Vieillot, 1817) | 11 | 2 |
| *Thraupis glaucocolpa* (Cabanis, 1850) | 11 | 5 |
| **Throchilidae** |  |  |
| *Leucippus fallax* (Bourcier, 1843) | 22 | 0 |
| **Tyrannidae** |  |  |
| *Elaenia parvirostris* (Pelzeln, 1868) | 20 | 0 |
| *Sublegatus arenarum* (Salvin, 1863) | 25 | 8 |
| **Total** | 527 | 216 |

Supplementary Table 2- Number of wild birds examined and infected by *Plasmodium* spp./ *Haemoproteus* spp. from Arid zone.
